# Reply: Allelic imbalance of TGFBR1 is not a major contributor to the genetic predisposition to colorectal cancer

**DOI:** 10.1038/bjc.2011.125

**Published:** 2011-04-26

**Authors:** L Valle, G Capellá, V R Moreno, G Rennert, S B Gruber

**Affiliations:** 1 Hereditary Cancer Program, Translational Research Laboratory, Catalan Institute of Oncology, IDIBELL, Av. Gran Vía 199-203, 08908 Hospitalet de Llobregat, Barcelona 08908, Spain; 2 Cancer Prevention and Control Program, Catalan Institute of Oncology, IDIBELL and University of Barcelona, Barcelona, Spain; 3 CHS National Cancer Control Center and Department of Community Medicine and Epidemiology, Carmel Medical Center and Bruce Rappaport Faculty of Medicine, Technion-Israel Institute of Technology, Haifa, Israel; 4 Department of Epidemiology, Department of Internal Medicine, and Department of Human Genetics, University of Michigan, Ann Arbor, MI, USA

Sir,

We appreciate the data and comments provided by Abadie *et al* (2011) regarding our recent report ‘No association between germline allele-specific expression of *TGFBR1* and colorectal cancer risk in Caucasian and Ashkenazi populations’ (Segui *et al*, 2011). They provide new compelling data corroborating our latest findings, which indicate that allele-specific expression (ASE) of *TGFBR1* does not confer an increased risk of colorectal cancer (CRC).

In their letter, Abadie *et al* criticise the lack of a detailed description of the subjects studied by Segui *et al* (2011). In this study, we show the characteristics of the informative cases and controls with ASE results that could be evaluated from the Ashkenazi Jewish and Caucasian cohorts (Table 1).

Abadie *et al* point out that the significant discrepancies observed among studies, mostly between the first (Valle *et al*, 2008) and the following reports (Guda *et al*, 2009; Carvajal-Carmona *et al*, 2010; Tomsic *et al*, 2010; Segui *et al*, 2011), may be partially attributed to the differences in the populations analysed. The sets of patients and controls analysed by Segui *et al* (2011) were obtained from one population-based and one hospital-based case–control study, with different selection criteria than the cases and controls selected by Abadie *et al.* Despite the differences, their results are in concordance with ours.

Abadie *et al* carried out the same methodological approach as Valle *et al* (2008). Therefore, to approximate the patients’ selection criteria in both the series in this study, we selected among the informative CRC cases studied in the original report those with: (1) CRC diagnosed before 61 years of age with a first-degree relative affected with CRC and (2) CRC diagnosed before 51 years of age (Valle *et al*, 2008). In this subset of cases, the average ASE value was 1.31 (range 0.79–3.95; *n*=63), very different from the average ASE value 1.07 (range 0.77–1.45; *n*=69) obtained by Abadie *et al* in CRC cases, also using the SNaPshot technology (PE Applied Biosystems, Foster City, CA, USA). This observation suggests that the difference in the study population is not the cause of discrepancies between the original and subsequent reports.

The results shown by Abadie *et al* indirectly support that SNaPshot is especially sensitive to RNA quality. Tomsic *et al* (2010) already noticed that high-quality RNA is essential for reproducibility of ASE. Although poor RNA quality may be the cause of inconsistent results with SNaPshot or pyrosequencing, the latter has proved to be a much more robust technique (Tomsic *et al*, 2010; Segui *et al*, 2011). The procedure of blood collection and processing carried out by Abadie *et al* ensured very high RNA quality, probably explaining why SNaPshot provided consistent values among SNPs and low s.d.'s between independent replicates, even for rs7871490, a SNP located in a complex repetitive stretch.

The letter by Abadie *et al* clarifies the role of ASE of *TGFBR1* in CRC susceptibility. Current evidence suggests that differences observed among studies are not directly attributable to study population or technique, provided high-quality RNA is used for allele expression experiments, and that allele-specific expression of *TGFBR1* is not a strong risk factor for CRC.

## Figures and Tables

**Table 1:** Characteristics of the informative cases and controls with ASE results studied by Segui *et al* (2011)

	**MECC**	**BCCS**
*CRC patients*	*n*=96	*n*=75
Age at CRC diagnosis (median±s.d.)	56±9.7	66±10.6
Gender	M: 46/93 (49%) F: 47/93 (51%)	M: 41/75 (55%) F: 34/75 (45%)
CRC family history (⩾1FDR with CRC)	10/92 (11%)	10/60 (17%)
Ethnicity	Ashkenazi	Caucasian
		
*Controls*	*n*=90	*n*=0
Age at blood collection (median±s.d.)	59±9.8	
Gender	M: 45/89 (51%) F: 44/89 (49%)	
CRC family history (⩾1FDR with CRC)	9/89 (10%)	
Ethnicity	Ashkenazi	

Abbreviations: BCCS=Bellvitge Colorectal Cancer Study; CRC=colorectal cancer; F=female; FDR=first-degree relative; M=male; MECC=Molecular Epidemiology of Colorectal Cancer; s.d.=standard deviation.
